# The use of Bayesian design in two trials in rare cancers

**DOI:** 10.1186/1745-6215-16-S2-P213

**Published:** 2015-11-16

**Authors:** Peter Dutton, Sharon Love, Ade Faleti, Bass Hassan

**Affiliations:** 1 Centre for Statistics in Medicine, University of Oxford, Oxford, UK; 2 NDORMS, Oxford, UK; 3 OCTO, Oxford, UK; 4 OMPI, SWDS, Oxford, UK

## 

Trials run in either rare diseases or rare subpopulations of common diseases are challenging in terms of identifying, recruiting and completing sufficient patients in a sensible time period. Moreover, the increasing emphasis on personalised and precision criteria for selection and endpoints during early drug development is changing the demands on trial designs because of a more limited number of patients.

We will discuss two active trials in bone sarcoma which use Bayesian methodologies. In designing these trials we needed to minimise the expected sample size whilst having acceptable properties. To do this a number of Frequentist and Bayesian approaches were considered and compare using Frequentist and Bayesian properties.

In the MEMOS trial, adaptations to Simon's two stage design to allow stopping early for efficacy and a Bayesian posterior predictive solution provide an efficient trial design.

In the LINES trial, a Bayesian posterior predictive approach allows co-primary endpoints to be accounted for effectively in this single arm trial. The results of the first interim analysis will be described.

## Funding

The research leading to these results has received funding from the European Union Seventh Framework Programme (FP7/2007-2013) under grant agreement n° 278742 (Eurosarc), NIHR funding from BRC and BRU and CRUK funding.

